# Dosage Compensation in *Drosophila*: Its Canonical and Non-Canonical Mechanisms

**DOI:** 10.3390/ijms231810976

**Published:** 2022-09-19

**Authors:** Yuri Y. Shevelyov, Sergey V. Ulianov, Mikhail S. Gelfand, Stepan N. Belyakin, Sergey V. Razin

**Affiliations:** 1 Institute of Molecular Genetics of National Research Centre “Kurchatov Institute”, Moscow 123182, Russia; 2 Institute of Gene Biology, Russian Academy of Sciences, Moscow 119334, Russia; 3 Faculty of Biology, M.V. Lomonosov Moscow State University, Moscow 119234, Russia; 4 Skolkovo Institute of Science and Technology, Skolkovo 143026, Russia; 5 A.A. Kharkevich Institute for Information Transmission Problems, Russian Academy of Sciences, Moscow 127051, Russia; 6 Institute of Molecular and Cellular Biology, Siberian Branch of Russian Academy of Sciences, Novosibirsk 630090, Russia

**Keywords:** dosage compensation, MSL complex, X chromosome, genome architecture, *Drosophila*

## Abstract

Dosage compensation equalizes gene expression in a single male X chromosome with that in the pairs of autosomes and female X chromosomes. In the fruit fly *Drosophila*, canonical dosage compensation is implemented by the male-specific lethal (MSL) complex functioning in all male somatic cells. This complex contains acetyl transferase males absent on the first (MOF), which performs H4K16 hyperacetylation specifically in the male X chromosome, thus facilitating transcription of the X-linked genes. However, accumulating evidence points to an existence of additional, non-canonical dosage compensation mechanisms operating in somatic and germline cells. In this review, we discuss current advances in the understanding of both canonical and non-canonical mechanisms of dosage compensation in *Drosophila*.

## 1. Introduction

Since heterogametic males of mammals and of most insects have an XY karyotype while females have an XX karyotype, there is a problem of imbalance of X-chromosomal gene expression between the two sexes. To eliminate this imbalance, special mechanisms of dosage compensation (DC) have emerged to ensure equal expression of X-linked genes in males and females. Early studies in *Drosophila melanogaster* with abnormal chromosome ploidy have shown that for several X-linked genes analyzed, amount (per cell) of the protein product in 3X;2A metafemales was almost the same as in 2X;2A (normal) females and in XY;2A (normal) males [1,2]. However, upon triple dose of autosomes (i.e., in XY;3A metamales, in 2X;3A intersexes, and in 3X;3A triploid females), X-linked genes generated approximately 1.5-fold more protein per cell [1,3,4,5,6]. Later, with the advent of transcriptomic studies, it became clear that in mammals, nematode, and fruit fly, the median expression of X-linked and autosomal genes in somatic cells is nearly equal [7,8,9,10]. Therefore, the main function of DC is to maintain a balance between X-chromosomal and autosomal gene expression independently of chromosome ploidy. However, DC mechanisms may not always work perfectly. For example, although X-linked gene expression is equalized between 3X;2A metafemales and normal females, autosomal gene expression in metafemales is decreased [2,11]. The imbalance between X-chromosomal and autosomal gene expression in metafemales may be responsible for their reduced viability [2].

In different organisms, DC is realized via somewhat similar principles [12]. In mammals, one of the two female X chromosomes is randomly heterochromatinized and inactivated [13,14,15], whereas gene expression from the other female X chromosome and from the single male X chromosome is upregulated [7,8,9,10]. In fruit fly, gene expression from the single male X chromosome is doubled [16]. In somatic cells of *Drosophila*, this twofold activation is partially mediated by the male-specific lethal (MSL) complex, which specifically binds to the male X chromosome and performs its H4K16 hyperacetylation, thus promoting transcription of the X-linked genes [17,18,19,20,21,22,23]. However, after knockdown of the key subunits of the MSL complex in male somatic cells, DC was not fully eliminated [24,25]. Moreover, in male germline cells, several MSL subunits are not expressed [26], but partial DC is functioning [7,9,27,28,29,30]. Furthermore, in early embryos, where the MSL complex is not yet recruited to the male X chromosome, transcripts derived from it are more abundant than transcripts derived from each female X chromosome [31]. Finally, in the metafemales with a 3X;2A genotype, the MSL complex is absent, whereas X-linked gene expression is decreased to become nearly the same as in normal females [11,32]. Based on these and other data, several research groups have supposed that, apart from the canonical DC, non-canonical mechanism(s) unrelated to the MSL complex activity may be responsible for partial DC in somatic and germline *Drosophila* cells [33,34,35]. Although several excellent reviews on DC in *Drosophila* have been published recently [36,37,38], they do not cover the latest results in this field. The present review summarizes the current state of our knowledge about DC phenomenon in *Drosophila*, focusing on the hypotheses explaining canonical and non-canonical mechanisms.

## 2. MSL Complex Mediates Canonical DC in *Drosophila*

The components of *Drosophila* MSL complex were initially identified in the genetic screening for male-specific lethality [18,39]. Later, genes carrying these mutations were cloned and characterized. As a result, the MSL complex is now known to consist of at least five proteins (MSL1, MSL2, MSL3, maleless (MLE), MOF) and either of *roX1* or *roX2* long non-coding RNAs [18,20,40,41,42,43,44,45,46,47,48,49,50]. Several chromatin proteins such as JIL-1 kinase, Megator (Mtor), Chromatin-linked adaptor for MSL proteins (CLAMP), Topoisomerase II (TopoII), and some others may represent additional components of the MSL complex, since they were shown to coimmunoprecipitate with the core MSL subunits [50,51,52,53,54,55,56]. Using a proximity ligation assay, it was found that core MSL proteins and *roX* RNAs as well as JIL-1, CLAMP, and TopoII are localized very close to each other on the male X chromosome [57]. Therefore, the latter proteins are physically associated with the MSL complex on the X chromosome, but have additional functions genome-wide. Of note, acetyltransferase MOF, the key component of the MSL complex, also belongs to the non-specific lethal (NSL) complex, which is bound with the housekeeping genes throughout the genome [50,58,59].

Brief characteristics of the core MSL complex components are provided below. The *roX1* and *roX2* RNAs are functionally nearly redundant since double mutations of *roX1* and *roX2* genes cause male lethality and loss of coverage by the MSL complex of the male X chromosome, whereas mutations of either *roX1* or *roX2* genes are phenotypically neutral [20,48,49,60]. However, lack of *roX1* or *roX2* RNAs affects expression of different gene sets, thus showing their incomplete interchangeability [61]. MSL1 interacts with MSL2, MSL3, and MOF, serving as a scaffold for the MSL complex assembly [62,63,64]. MSL3 contains a chromodomain which recognizes the H3K36me3 mark of active gene transcription [65,66,67]. Due to this property, it is supposed to be responsible for the MSL complex occupancy along the bodies of expressed genes with the enrichment at their 3’-ends [68,69]. MLE is a RNA/DNA-helicase [70] necessary for incorporation of *roX* RNAs into the MSL complex [20,71]. MOF is an H4K16ac-specific acetyltransferase [18,19,21]. Among other MSL components, MSL2 is the only male-specifically expressed protein. It contains a DNA-binding (CXC) domain which participates in the MSL complex targeting of the male X chromosome [43,45,46,72,73]. MSL2 also contains the RING finger domain, which mediates E3 ubiquitin ligase activity. Apart from other functions, MSL2 maintains the correct stoichiometry of the MSL subunits by their ubiquitylation and further proteasome-dependent degradation, if the stoichiometry is unbalanced [74].

The current model explaining the delivery of the MSL complex to virtually all expressed genes on the male X chromosome includes at least two stages [75]. At the first stage, the MSL complex binds to approximately two hundred high-affinity/chromatin entry sites (HAS/CES) on the X chromosome [76,77]. At the next stage, it spreads from HAS/CES to the nearby expressed genes due to an association of MSL3 subunit with the H3K36me3 modification over these genes [65,66,67], although recently, the role of H3K36me3 in this process has been questioned [78]. It was suggested that the MSL subcomplex consisting of only MSL1 and MSL2 [79,80,81] is sufficient for HAS/CES recognition, since *msl3*, *mle*, and *mof* null and *roX1* and *roX2* double-null mutant males retain occupancy of these sites by the MSL1-MSL2 subcomplex [76,77,82]. Each HAS/CES contains from one to several copies of a 21-bp GA-rich sequence motif called MSL recognition element (MRE) [76,77], yet only a small fraction of all MREs are localized within HAS/CES [83]. This raises the question of whether MREs are the key determinants for X chromosome recognition. The CXC domain of the MSL2 subunit was shown to bind some MREs, but the specificity of these interactions was low [72,73]. Recently, using experiments with in vitro binding of the MSL2 protein with purified genomic DNA, it was found that the CXC domain with much higher specificity recognizes the PionX (pioneering on the X) sites, which represent the subset of HAS/CES carrying additional sequences at the 5’-ends of MREs [84]. Moreover, upon artificial initiation of the MSL complex assembly in the female Kc167 cell line, the MSL complex binds PionX sites earlier than other MREs [84]. Therefore, PionX sites may potentially recruit the MSL complex to the X chromosome independently from other factors. MREs are also recognized by DNA-binding protein CLAMP, which is involved in the MSL recruiting to HAS/CES [85,86]. However, apart from HAS/CES, numerous CLAMP-bound regions were revealed on the X chromosome and on autosomes [85,86]. Yet, the density of GA-rich sequence motifs is higher within HAS/CES than within other CLAMP-bound regions, which correlates with higher CLAMP occupancy at these sites [87]. Hence, X chromosome has DNA sequence features which discriminate it from autosomes. Recent studies have shown that MSL2 and CLAMP physically interact with each other, and that the CXC and CLAMP-binding domains of MSL2 are both necessary for the MSL complex targeting the X chromosome [55,88]. Moreover, upon CLAMP depletion in male S2 cells, the MSL complex deoccupies HAS/CES, including the PionX sites [55]. Therefore, it was suggested that cooperative binding of the MSL2 and CLAMP to different MREs located within the same HAS/CES determines the MSL complex occupancy of these X chromosomal sites in males [55,88]. In addition, CLAMP was shown to make chromatin more open, and its binding to MREs may facilitate MSL2 binding [55,89].

Since *roX1* and *roX2* gene positions on the X chromosome coincide with two HAS/CES, *roX* RNAs were supposed to be incorporated in the incomplete MSL complex at the sites of their nascent transcription. Apart from this, *roX* RNAs may be incorporated in the MSL complex in the nucleoplasm (before it is bound with chromatin), or already on chromatin of other HAS/CES [49,75,90]. For inclusion in the complex, RNA/DNA-helicase MLE unwinds stem-loop structures in the *roX* RNAs, making them accessible for interactions with the MSL2 subunit [91,92,93,94,95]. The inclusion of *roX* RNAs in the MSL complex triggers its spreading from HAS/CES to the nearby regions [20,71,75]. For example, autosomal *roX* transgenes create new HAS/CES at the sites of insertions from which the MSL complex spreads bi-directionally at hundreds of kilobases into autosomal chromatin [75]. It remained unclear how *roX* RNAs were involved in this process. Recent work has shed light on this puzzle. It was found that MSL2 contains intrinsically disordered regions (IDRs) at the C-terminus, which are able to form phase-separated droplets with either *roX2* or, less efficiently, with *roX1* RNAs [96]. The increased concentration of *roX* RNAs, MSL2, and other MSL subunits within these droplets, which envelope HAS/CES and nearby chromatin, may facilitate binding of the MSL complex to the low-affinity sites and its further spreading to H3K36me3-modified chromatin of active genes. Additionally, the acetyltransferase activity of MOF is necessary for the MSL spreading, since the *mof* mutation specifically eliminating this activity also blocks the spreading [71].

There are somewhat contradictory results from different groups regarding the involvement of *roX* RNAs in the initial binding of the MSL1–MSL2 subcomplex to HAS/CES [84,96]. One argument in support of *roX* RNA involvement in the MSL targeting HAS/CES is that the replacement of the C-terminal domain of *Drosophila* MSL2, carrying IDRs, with its counterpart from mouse MSL2, lacking IDRs, results in the loss of binding of the hybrid MSL complex to HAS/CES [96]. It should be noted, however, that the replaced region in *Drosophila* MSL2 also contained the CLAMP-binding domain, which participates in the cooperative recognition of HAS/CES by MSL2 and CLAMP [55,88]; this domain may be missing in the mouse MSL2. An additional argument is the observed positive correlation between *roX2* expression level and MSL2 occupancy at HAS/CES [96]. Furthermore, *roX2* overexpression partially restored the MSL complex recruiting to the X chromosome upon simultaneous mutations in the CXC and CLAMP-binding domains of MSL2 [97]. The idea that *roX* RNAs may enhance MSL2 binding to HAS/CES has gained support in the studies of MSL targeting the male X chromosome in *D. virilis* [98]. In this species, the role of CLAMP is less essential than in *D. melanogaster*, while *roX* RNAs are more instructive in specifying MSL2-binding sites. The authors suppose that interactions between *roX* RNA and MSL2 may allosterically modulate MSL2 conformation, thus enhancing MSL2 binding capacity towards HAS/CES.

Recent works analyzing genome architecture have uncovered additional layers of regulation of the MSL complex recruiting and spreading within the male X chromosome [99,100,101,102]. It appears that HAS/CES frequently interact with each other in the three-dimensional (3D) space of the nucleus [99]. Strikingly, these interactions are mainly non sex-specific, as they were almost identical in male and female cell lines [99]. Moreover, the interactions between HAS/CES were not altered upon depletion of either MSL2 or MSL3 subunits [99]. Therefore, the specific 3D conformation of the male X chromosome is not mediated by the MSL complex. However, this conformation is important for MSL propagation. Indeed, the MSL complex was detected on polytene chromosomes at a site which was separated from the site of autosomal *roX2* transgene insertion by ~2.6 Mb, with no detectable binding within a ~1.7-Mb region in between these sites [99]. Furthermore, upon artificial induction of the MSL complex assembly in the female Kc167 cell line, domains of activated gene expression were interspersed with the domains where gene expression was not altered [100]. These data point to the discontinuous mode of spreading of the MSL complex, likely occurring in 3D. Thus, HAS/CES form interaction hubs necessary for efficient 3D spreading of the MSL complex.

A more detailed analysis of genome architecture in sex-sorted embryos revealed some differences in the X chromosome organization in males and females [100,101]. The interactions within the active compartment of the X chromosome appeared to be slightly more frequent in males than in females, which correlates with higher H4K16ac levels in the male X chromosome [100]. 3D interactions between HAS/CES constitute a fraction of all reinforced interactions within the male X chromosome [100,101]. It remains unclear whether the enhanced interactions between HAS/CES located in the active chromatin are the manifestation of the increased contact frequency within the active compartment as a whole, or whether they are mediated by additional factors. A recent study [102] has provided some clues to answer this question. The interactions of HAS/CES with other active regions on the X chromosome appeared to be more frequent than between active regions without HAS/CES. Furthermore, upon depletion of CLAMP in male S2 cells, the interactions between active regions (including HAS/CES) on the X chromosome were more weakened than between active regions on autosomes [102]. CLAMP is capable for homodimerization [103] and physically associates with architectural proteins, including CP190 [104]. Taken together, these findings indicate that CLAMP not only promotes binding of the MSL2 with HAS/CES, but is also responsible for 3D interactions of different HAS/CES with each other as well as with other active regions on the X chromosome [102], thus creating a “hard-wired” [101] X chromosome conformation favorable for the MSL complex spreading.

How does the MSL complex perform transcriptional activation of the male X-linked genes? Almost 30 years ago, it was found that polytene X chromosome of *Drosophila* males differs from autosomes and from X chromosomes of females by a drastic increase in H4K16 acetylation [105,106]. Later studies using chromatin immunoprecipitation (ChIP) revealed two distinct types of averaged H4K16 acetylation profiles along active genes in somatic cells. The profile of H4K16 acetylation over genes located on the autosomes and female X chromosomes has a peak of increased acetylation at their 5’-ends, whereas the profile over male X-linked genes goes higher and is nearly evenly distributed along gene bodies with some elevation towards their 3′-ends. Moreover, H4K16 acetylation spreads further up- and downstream of genes [22,23,107,108]. Histone acetylation was associated with gene activation [109], and H4K16 acetylation was shown to result in chromatin decondensation by disrupting the interactions between histone H4 tail on one nucleosome and the acidic patch interface formed by the H2A–H2B dimer on another nucleosome [110,111]. Based on these data, a model arose wherein acetyltransferase MOF, introducing this modification, performs X chromosome-specific H4K16 acetylation, which promotes transcription of the male X-linked genes [18,19,21,112]. Several lines of evidence support this model. It is supported by transcriptomic studies which have shown partial loss of equilization of expression of X-linked genes in males and females upon depletion of different MSL subunits [24,25]. Moreover, flies with H4K16 to H4R16 substitution or with the mutations in the *mof* gene demonstrate the male-specific lethality [18,113,114], similarly to the *msl* mutants, thus pointing to the causative role of MOF and H4K16 acetylation in the canonical DC. Finally, upon lack of maternally supplied H4K16ac, caused by MOF depletion in oocytes, DC was compromised in late embryos [115].

Whether the increased H4K16 acetylation facilitates initiation or elongation of transcription, or both, has been a matter of debate, since when using ChIP-seq, the 1.2-fold increase in the Pol II occupancy was revealed at both the promoters and gene bodies of the X-linked genes compared to autosomal genes [116,117], whereas when using global run-on or direct nascent RNA sequencing, the 1.4-fold increase was revealed only over gene bodies [22,118]. Importantly, depletion of MSL2 subunit reduces Pol II occupancy on the X-linked genes to the level which was detected on the autosomal genes [22,116], thus indicating that higher Pol II levels were mediated by the MSL complex activity. Taken together, these results indicate that H4K16 acetylation deposited by MOF (as a constituent of the MSL complex) over X-linked genes may facilitate Pol II targeting the promoters and/or Pol II transition to the transcriptional elongation. The question of how the ~1.4-fold increase in Pol II occupancy over X-linked genes relative to autosomal genes is converted to the 2-fold increase in their steady-state transcript levels remains open.

If the increased H4K16 acetylation enhances transcription of the X-linked genes, then what is the mechanism that restricts this enhancement to only a 2-fold value? Recent work has shown that nucleoporin Mtor is involved in this process [56]. Mtor was earlier shown by ChIP to cover 75% of the male X chromosome in the wide domains carrying a high level of H4K16 acetylation [54], thus pointing to its possible role in DC. Moreover, Mtor was coimmunoprecipitated with the acetyltransferase MOF responsible for this acetylation [50,56]. Based on these results, as well as on the microarray expression data upon Mtor depletion, it was supposed that Mtor participates in DC by facilitating transcription of the X-linked genes in *Drosophila* males [54]. However, contrary to this hypothesis, RNA-seq analysis, performed later, has unambiguously demonstrated that the majority of active genes on the X chromosome begin to transcribe more strongly upon depletion of Mtor in male S2 cells [56]. Importantly, this activation was mitigated upon simultaneous MOF depletion [56]. Therefore, the role of Mtor in DC is not to enhance, but rather to restrict the hyperactivation of transcription of dosage-compensated genes. Interestingly, in salivary gland polytene chromosomes, lack of Mtor does not impair X-chromosomal localization of either MOF or H4K16 acetylation [56], thus showing that Mtor normally attenuates transcription within highly acetylated chromatin of the X-linked genes. It regulates Pol II transition from hypophosphorylated to Ser-5-phosphorylated state, capable for initiation of transcription [56].

**Figure 1:**
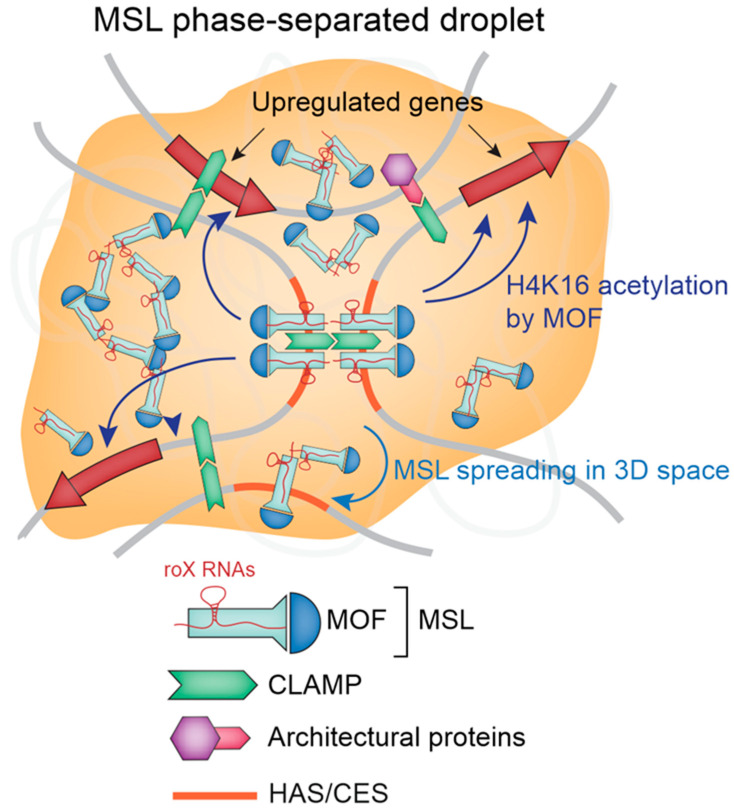
Schematic illustrating mechanisms involved in canonical DC. See text for details.

Altogether, these studies draw the complicated picture of how the MSL complex realizes its mission in the canonical DC (Figure 1). This picture includes cooperative binding of MSL2 and CLAMP to HAS/CES, the fine-tuning of their binding specificity by *roX* RNAs, the formation of phase-separated droplets induced by binding between *roX* RNAs and MSL2, the creation of “hard-wired” 3D organization of the X chromosome shaped by CLAMP, the spreading of the MSL complex in 3D from HAS/CES to the active X-linked genes due to MSL3 association with their H3K36me3 mark, the stimulation of transcription of the X-linked genes by MOF (which deposits H4K16 acetylation along gene bodies), and, finally, the restriction of transcriptional activation to a twofold value by Mtor.

## 3. Non-Canonical Mechanisms of DC in *Drosophila*

The canonical DC mechanism which relies on MSL complex activity does not ensure twofold upregulation of the X-linked genes. For example, depletion of MSL complex subunits in the male somatic S2 or SL-2 cells did not lead to complete loss of DC, but only ~1.4-fold downregulated X-linked gene expression [24,25]. Furthermore, in early *Drosophila* embryos, the MSL complex is not yet bound with the X chromosome, whereas partial DC is revealed [31]. Additionally, in male early germline cells, several components of the MSL complex (MSL1, MSL2, MSL3, MLE, *roX1* and *roX2* RNAs) are absent or only barely expressed, and H4K16 acetylation is equally represented on the X chromosome and on autosomes [26,29]. Nevertheless, the vestiges of DC (i.e., partial equilization of X and autosomal gene expression) were detected in these cells. However, based on RNA expression studies, the magnitude of DC has been claimed to vary from complete absence to full compensation, depending on the study [7,9,27,28,119]. Recently, the existence of DC in the male germline was confirmed using the transgenic approach. Median testes expression of one dose of early germline-specific reporter was found to be 1.8-fold higher upon its random insertions in the X chromosome, as compared to autosomes [30]. Collectively, these data support the idea that non-canonical DC functions in both male somatic and germline cells and that it may account for roughly half of the activation of X-linked genes in both cell types [7,25,30].

What might be the nature of non-canonical mechanisms? It was supposed that non-canonical DC may be mediated by an “inverse dosage” effect [35] or by “buffering” [34], which are characterized by some propensity to restore normal gene expression levels despite changes in gene dose. Numerous cases of these effects have been documented when gene dose was altered due to segmental monosomy or trisomy [25,120]. It was hypothesized that these effects may be caused by the imbalance in stoichiometry of the protein complexes, which by feedback loop would result in altered transcription of the corresponding genes. If genes encoding transcription factors and their gene targets are located in the same aneuploid genome region, this may also lead to buffering [34]. However, it is unclear how the inverse dosage effect or buffering may account for the coordinated changes of gene expression in the entire aneuploid chromosomes, since in this case, a multitude of genes on different chromosomes should be dysregulated in the opposite directions. Therefore, both effects, when applicable to the entire chromosomes, have so far only a descriptive nature which does not have any obvious molecular explanation.

**Figure 2:**
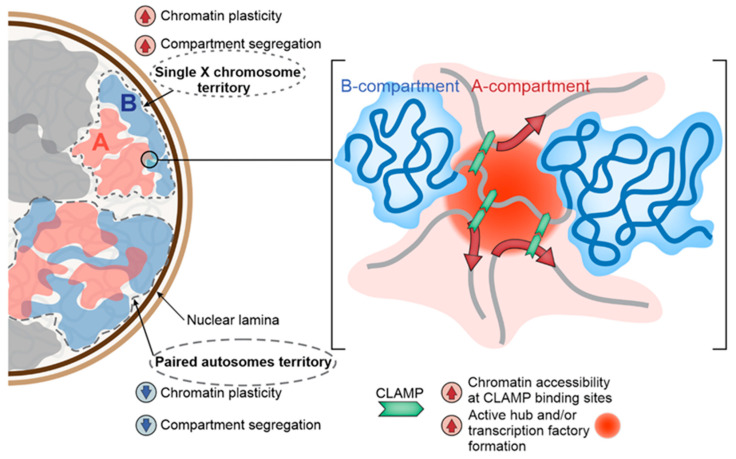
Schematic illustrating mechanisms involved in non-canonical DC. See text for details.

*Drosophila* spermatogenesis is a useful model for the analysis of non-canonical DC mechanisms, since male germline cells have the same karyotype as male somatic cells, but the MSL complex does not operate in these cells [26]. Recent analysis of genome architecture in early germline cells of testes [30] provides an opportunity to compare 3D organization of the genome in male somatic and germline cells. In early germline cells of males, the interactions within the active X-chromosomal compartment were found to be enhanced compared to interactions within an autosomal one [30]. A similar picture was observed in male somatic cells, where HAS/CES were shown to interact stronger with each other and with active regions of the X chromosome compared to autosomes [99,100,101]. Such an organization may facilitate transcription by promoting the MSL complex delivery from HAS/CES to the X-linked genes in somatic cells [99], or by delivery of X-linked genes to the active hubs or transcription factories in germline cells [30]. What is the nature of the “hard-wired” organization of the X chromosome in male germline cells? The DNA sequence content of the X chromosome, particularly its enrichment with CLAMP-bound motifs [87], may determine this organization. CLAMP, which is able to bind with the GA-enriched HAS/CES mainly independently from the MSL complex [86] and is capable for dimerization [103], is actively expressed in early germline cells of testes [29]. Therefore, CLAMP may play an important role in the non-canonical DC in these cells by making chromatin around its binding sites more “open” and accessible [89,101], and by promoting long-range chromatin interactions within the active compartment of the X chromosome [102] (Figure 2).

Another idea is that the enhanced interactions within the active compartment of the male X chromosome may be achieved due to increased plasticity of the single X chromosome stemming from the lack of its paired homologue. Indeed, computer modeling has shown that the absence of a paired homologue makes the X chromosome less rigid and more prone to interactions within the active compartment, which may facilitate transcription [101]. In support of this idea, the median expression of early germline-specific reporter in testes was slightly higher when transgene was inserted in the unpaired as compared to the paired autosomes [30].

One more hypothesis is that binding of peripheral chromatin with the nuclear lamina may enhance chromatin interactions between active regions located in the nuclear interior. It was shown that in early germline cells of testes, X chromosome is more strongly bound with the nuclear lamina than autosomes [30]. This effect may be mediated by the competition between different peripheral chromatin regions, located within a chromosome territory, for binding with the nuclear lamina which underlies this chromosome territory [30,121,122]. Paradoxically, the stronger binding of the single X chromosome with the nuclear lamina correlates with higher expression levels of the X-linked genes [7,9,27,28,29,30] and with the enhanced spatial segregation of active and inactive compartments in the X chromosome [30]. Since interactions within the active compartment became slightly weaker upon loss of binding of inactive peripheral chromatin with the nuclear lamina after lamin depletion in the S2 cell line [123], it is reasonable to assume that it is the attachment of peripheral chromatin to the nuclear lamina that makes the active compartment of the male X chromosome more spatially segregated from the inactive one [30]. The spatial segregation may favor the formation of transcription factories or active hubs in the active compartment, thus promoting transcription of the X-linked genes (Figure 2).

It should be mentioned that the hypotheses regarding non-canonical mechanisms of DC are not mutually exclusive. However, the question of which of them are actually realized in *Drosophila* cells needs further studies.

## 4. Conclusions and Outlooks

Despite several decades of research on DC in various organisms, many questions still remain unresolved. *Drosophila* is one of the first organisms where these mechanisms have been studied and where many important results clarifying DC have been obtained. Nevertheless, while the canonical DC has become more clearly interpreted over time, the non-canonical mechanisms are far from being understood. There are many hypotheses, but they were not tested directly. In light of the similarity of DC mechanisms in *Drosophila* and humans, studies on the *Drosophila* model become more relevant. The advent of modern technologies opens avenues for future research in this field.

## Abbreviations

3D	Three-dimensional
CES	Chromatin entry sites
ChIP	Chromatin immunoprecipitation
CLAMP	Chromatin-linked adaptor for MSL proteins
DC	Dosage compensation
H3K36me3	Histone H3 tri-methylated at Lys 36
H4K16ac	Histone H4 acetylated at Lys 16
HAS	High-affinity sites
LAD	Lamina-associated domain
MLE	Maleless
MOF	Males absent on the first
MRE	MSL recognition element
MSL	Male-specific lethal
Mtor	Megator
NSL	Non-specific lethal
PionX	Pioneering on the X
Pol II	RNA polymerase II
TopoII	Topoisomerase II
